# Tumor Mouse Model Confirms MAGE-A3 Cancer Immunotherapeutic As an Efficient Inducer of Long-Lasting Anti-Tumoral Responses

**DOI:** 10.1371/journal.pone.0094883

**Published:** 2014-05-15

**Authors:** Catherine Gérard, Nathalie Baudson, Thierry Ory, Jamila Louahed

**Affiliations:** GlaxoSmithKline Vaccines, Rixensart, Belgium; Leiden University Medical Center, Netherlands

## Abstract

**Purpose:**

MAGE-A3 is a potential target for immunotherapy due to its tumor-specific nature and expression in several tumor types. Clinical data on MAGE-A3 immunotherapy have raised many questions that can only be addressed by using animal models. In the present study, different aspects of the murine anti-tumor immune responses induced by a recombinant MAGE-A3 protein (recMAGE-A3) in combination with different immunostimulants (AS01, AS02, CpG7909 or AS15) were investigated.

**Experimental Design and Results:**

Based on cytokine profile analyses and protection against challenge with MAGE-A3-expressing tumor, the combination recMAGE-A3+AS15 was selected for further experimental work, in particular to study the mechanisms of anti-tumor responses. By using MHC class I-, MHC class II-, perforin-, B-cell- and IFN-γ- knock-out mice and CD4^+^ T cell-, CD8^+^ T cell- and NK cell- depleted mice, we demonstrated that CD4^+^ T cells and NK cells are the main anti-tumor effectors, and that IFN-γ is a major effector molecule. This mouse tumor model also established the need to repeat recMAGE-A3+AS15 injections to sustain efficient anti-tumor responses. Furthermore, our results indicated that the efficacy of tumor rejection by the elicited anti-MAGE-A3 responses depends on the proportion of tumor cells expressing MAGE-A3.

**Conclusions:**

The recMAGE-A3+AS15 cancer immunotherapy efficiently induced an antigen-specific, functional and long-lasting immune response able to recognize and eliminate MAGE-A3-expressing tumor cells up to several months after the last immunization in mice. The data highlighted the importance of the immunostimulant to induce a Th1-type immune response, as well as the key role played by IFN-γ, CD4^+^ T cells and NK cells in the anti-tumoral effect.

## Introduction

Ever since William Coley’s observations in the 19th century that cancer may be treated by mobilizing the patient’s own immune system, the ultimate goal for cancer immunologists has been to reproducibly achieve this in patients. The mutated aberrant proteins, re-activated or over-expressed in tumor cells, represent potential “tumor antigens” that can be targeted by the immune system [1]–[3].

Aberrant gene promoter demethylation is an important mechanism by which the expression of normally silent genes is re-activated in tumor cells. This is the case for the *MAGEA* family of genes that are normally expressed during embryonic life [4] and in the placenta [5], [6], but are silent in normal adult tissues, except in the germline cells of the testis [5].

MAGE-A3, a member of this MAGE-A family, is an attractive tumor antigen, as i) it is almost exclusively expressed in tumors, eliminating the risk of mounting an active immune response against normal tissues (germ cells of the testis are the only normal cells expressing MAGE-A3, but they are devoid of classical HLA class I–II molecules and hence have no antigen presentation capabilities, which exclude the development of immune-related toxicity upon MAGE-A3 immunotherapy), ii) it is expressed in many different cancer types, and iii) it is naturally immunogenic, as CD8^+^ T lymphocytes specific for MAGE-A3 were found to infiltrate tumor sites in melanoma patients [7].

Clinical data generated over the last decade using different immunotherapeutic approaches showed that delivering MAGE-A3 as a purified recombinant protein formulated with an immunostimulant may be a promising approach [8]–[11]. Nevertheless, despite encouraging results, many issues remain to be solved to further improve MAGE-A3-specific immunotherapy. In particular, improving the MAGE-A3-immunostimulant combination to induce long lasting anti-tumor immune responses remains essential. In addition, the precise mechanisms and key immune effectors leading to tumor rejection are not known, and no clear immune correlate for clinical efficacy has yet been determined. Nor is it known to which extent the focal pattern of MAGE-A3 expression within a tumor can limit clinical efficacy. Such questions and hypotheses cannot reasonably be addressed in clinical trials, due to the long duration and limited number of patients. Therefore, pre-clinical studies remain essential to guide the clinical development of MAGE-A3-specific immunotherapy.

We addressed some of these questions in the present study. In a first series of experiments, mice were immunized with recombinant MAGE-A3 (recMAGE-A3) formulated with different immunostimulants: AS01, AS02, AS15 or CpG7909. AS15 was selected from this panel for further investigation, due to its capacity to drive the immune system towards a Th1-type immune response and the resulting anti-tumor activity against MAGE-A3-expressing tumor cells. Mice were therefore immunized with the selected recMAGE-A3+AS15 formulation in another series of experiments to evaluate i) the key effectors involved in the anti-tumor activity, ii) the influence of booster injections and iii) the impact of tumor heterogeneity -i.e. the proportion of tumor cells expressing MAGE-A3- on this anti-tumor activity.

## Materials and Methods

### Ethics Statement

Experiments were carried out in GlaxoSmithKline Vaccines laboratories or by GlaxoSmithKline staff at Armand Frappier Institute (IAF - Canada). Animal studies disclosed in this manuscript were ethically reviewed and approved by the GlaxoSmithKline Vaccines’ Belgian ethical Committee for Animal Experimentation or by the Ethics Committee of the IAF. They were conducted in accordance with European Directive 2010/63/EU, the CCAC standards (Canadian council for Animal Care), and the GlaxoSmithKline Vaccines Policy on the Care, Welfare and Treatment of Animals. Both GlaxoSmithKline Vaccine facility and IAF are AAALAC (Association for Assessment and Accreditation of Laboratory Animal Care) accredited. All efforts were made to minimize suffering: tumors exceeding a maximum allowable size of 17 mm×17 mm, ulceration, tumor necrosis, convulsion, morbidity and circling behavior were conditions requiring euthanasia by intra-peritoneal injection with barbituric acid derivative (overdose).

### Antigen Description, Production and Purification

The fusion protein ProtD–MAGE-A3-His, also abbreviated recMAGE-A3, contains the first 127 residues of protein D derived from *Haemophilus influenzae* at its N-terminus to improve the protein expression in a bacterial system, and a sequence of histidine residues at its C-terminus to facilitate the fusion protein purification.

The production of recMAGE-A3 was performed in the *Escherichia coli* strain AR58, as described previously [11]. Another recombinant MAGE-A3 protein, consisting of the first 314 amino acids of MAGE-A3 followed by 6 histidine residues, was produced in baculovirus [11]. This protein, referred to as bacMAGE-A3, was used in the monitoring of the immune responses.

### Description of the Immunostimulants

AS02 consists of an oil-in-water emulsion containing 3-*O*-desacyl-4′-monophosphoryl lipid A (MPL, GlaxoSmithKline Vaccines, Rixensart, Belgium), a Toll-like receptor (TLR)-4 agonist, and QS-21 (*Quillaja saponaria* Molina fraction 21, Antigenics Inc, a wholly owned subsidiary of Agenus Inc., Lexington, MA, USA), which is a molecule of the saponin family [12]. AS01 is an Adjuvant System containing MPL, QS-21 and liposome. AS15 contains MPL, QS-21, liposome, and the TLR-9 ligand CpG7909 (synthetic oligodeoxyribonucleotides [ODNs] containing unmethylated CpG motifs; herein referred to as CpG).

### Mouse Strains and Immunizations

C57BL/6 or CB6F1 (hybrid between C57BL/6 and BALB/c) female mice (6–8 week-old) were purchased from Harlan (Horst, The Netherlands) and kept in specific pathogen-free conditions.

Mice were usually injected 2 or 4 times intra-muscularly at 2-week intervals with 1 or 10 µg of recMAGE-A3 in 50 µl of immunostimulant.

To study long-term protection, mice received 2 injections of either recMAGE-A3+AS15 or phosphate-buffered solution (PBS) at 2-week intervals. Eight weeks after the second immunization, the animals were challenged with a TC1-MAGE-A3 tumor (see description of the tumor cells below; *Tumor models and challenges*). On Day 150, 80 days after tumor challenge, tumor-free animals from the recMAGE-A3+AS15 group were randomized and allocated to two groups. One group received four booster injections of recMAGE-A3+AS15 at a 4-week interval and the other group received injections of PBS following the same schedule. Thirty days after the last injection, mice underwent a tumor challenge in the same flank, and tumor growth was monitored during 46 days (up to Day 319). Additionally, tumor cells were injected into a group of ten PBS-immunized mice, as a positive control for tumor growth.

To assess the role of IFN-γ, perforin and MHC class I or II molecules in tumor protection following MAGE-A3 immunotherapy, immunodeficient mice were used with the same immunization schedules as described above. The following strains were purchased from the Jackson Institute: IFNγ-knocked out (KO) mice (B6.129S7-Ifngtm1Ts/J), MHC class I-KO (B6.129P2-b2mtm1Unc), MHC class II-KO (B6.129S2-H2-dIAb1-Ea00451), B cell-KO (B6.129S2.IgHmTm1Cgn) and perforin-KO mice (C57BL/6-Prf1 tm1Sdz/J).

To assess the potential role of T cells, recMAGE-A3-immunized C57BL/6 mice were depleted of CD4^+^ or CD8^+^ T cells by injecting 0.5 mg rat anti-mouse antibodies (GK1.5 [TIB-207 from ATCC] and 2.43 [TIB-210 from ATCC], respectively) one week before the tumor challenge and then weekly during the course of the experiment. NK cell depletion was achieved by injecting the anti-Asialo GM1 antibody (Cedarlane) twice a week starting at day 49 (i.e. 7 days before tumor challenge) and until the end of the experiment (0.1 ml per injection). Depletions were verified by flow cytometry (data not shown). Control antibodies with similar isotypes to the depleting antibodies were used as negative controls.

### Tumor Models and Challenges

TC1-MAGE-A3 cells are murine tumor cells genetically modified to express human MAGE-A3. TC1 tumor cells (obtained from Dr T. Wu, John Hopkins University) are interesting as they recapitulate the different steps leading to a tumorigenic cell line. Originally, the TC1 tumor cell line was generated from C57BL/6 primary lung epithelial cells immortalized by transfection of the *Hpv-16 e6* and *e7* genes, and transformed with an activated *Ras* oncogene [13]. These cells were transfected with a pcDNA3 plasmid containing *MAGEA3* cDNA and the *zeocin* selection gene. Clones resistant to zeocin treatment were tested for *MAGEA3* expression by RT-PCR and for MHC class I expression by flow cytometry (data not shown). The best clone showing reproducible tumorigenicity in mice was chosen.

For each challenge, the animals received a subcutaneous injection of 2×10^6^ TC1-MAGE-A3 cells (200 µl in the flank). Individual tumor growth was recorded twice a week, by measuring the product of the 2 main diameters of the tumor during the monitoring phase, starting 7 days after the day of challenge. Mice were sacrificed during the study when the tumor size reached 289 mm^2^. In such case, the value of the last measurement obtained prior to sacrifice was carried forward to the next time point(s).

To determine whether a threshold percentage of MAGE-A3-expressing tumor cells is needed to elicit tumor rejection by the immune system, PBS-sham-immunized mice and mice immunized with recMAGE-A3+AS15 were challenged with TC1 parental cells (100% MAGE-A3-negative cells), TC1-MAGE-A3 cells (100% MAGE-A3 expressing cells), or different ratios of TC1/TC1-MAGE-A3: i.e. 10/90, 50/50 or 90/10%, respectively.

### Cytokine Production

Isolated mouse splenocytes were cultured in the presence of 1 µg/ml bacMAGE-A3. After 72 h, the concentrations of IL-2, IL-4, IL-5, IFN-γ and TNF-α in the supernatants were measured by cytometric bead array (CBA, Pharmingen cat n° 551287), according to the manufacturer’s instructions.

### Intracellular Cytokine Staining and Flow Cytometry

Peripheral blood mononuclear cells isolated from immunized animals were stimulated *in vitro* in 96-round bottom well plates with either medium (no stimulation) or a pool of fifty-seven 15 mer peptides overlapping by 10 amino acids, covering the entire sequence of MAGE-A3 (1 µg/ml for each peptide), in a final volume of 200 µl of RPMI, 5% fetal calf serum (FCS) containing rabbit anti-mouse anti-CD49d and anti-CD28 antibodies (Becton Dickinson, BD n° 553154 and n° 553295 respectively; final concentration: 1 µg/ml each). After 2 h of incubation at 37°C, the secretion of cytokines was blocked by the addition of 50 µl brefeldin (Golgi Plug, BD n° 555029: 1/1000 in RPMI 5% FCS). Cells were transferred to a 96-conical bottom well plate, centrifuged and washed with 250 µl PBS containing 1% FCS (FACS buffer). The cell pellets were incubated for 10 min at 4°C in the presence of rat anti-mouse CD16/CD32 (2.4G2, BD n° 553142; 0.5 mg/ml) to block Fcγ receptors. CD4^+^ and CD8^+^ T cells were stained for 30 min at 4°C by adding 50 µl phycoerythrin-labeled rat anti-mouse CD4 monoclonal antibody (BD n° 556616) or peridinin chlorophyll protein-labeled rat anti-mouse CD8 monoclonal antibody (BD n° 553036). After a washing step, the cells were fixed in 200 µl of cytoFix-cytoPerm solution (BD n° 554722) for 20 min at 4°C and permeabilized by adding permWASH solution (BD n° 554723). After centrifugation, cells were incubated 2 h at 4°C with 50 µl of a mix of allophycocyanin-labeled anti-IFN-γ (BD n° 554413). Cells were washed, centrifuged and resuspended in FACS buffer before flow cytometry analysis (LSR2 from BD). Gating was done on T cells, and a total of approximately 20,000 CD4^+^ T cells were acquired. The data were expressed as percentages of MAGE-A3-specific IFN-γ-producing CD4^+^ or CD8^+^ T cells amongst the total population of CD4^+^ or CD8^+^ T cells, respectively, after subtraction of control medium value.

### Statistical Analyses

Cytokine analyses were performed using an ANOVA with group as factor after log-transformation of the data. For other analyses, the statistical model was a repeated ANOVA with group, time and group-by-time interaction as factors; the correlation between two measurements from the same mice is assumed to be autoregressive, i.e. correlations decline exponentially with time. Variances were assumed to be different across groups but identical across time points. Comparisons of the mean tumor sizes were made at the last time point.

## Results

### Immune and Anti-tumor Responses in Mice Immunized with recMAGE-A3 Combined with Different Immunostimulants

After four immunizations of C57BL/6 mice with recMAGE-A3, alone or formulated with an immunostimulant (AS01, AS02, CpG or AS15), both humoral and cellular immune responses were assessed. The antibody response was low after immunization with recMAGE-A3 alone, compared with immunization with recMAGE-A3 formulated with an immunostimulant (Figure S1). The humoral responses induced by recMAGE-A3 formulated with different immunostimulants were considered equivalent, irrespective of the immunostimulant. Similarly, no major differences were observed between the immunostimulants in their ability to induce T-cell responses as evaluated by lympho-proliferation experiments (Figure S2).

**Figure 1:**
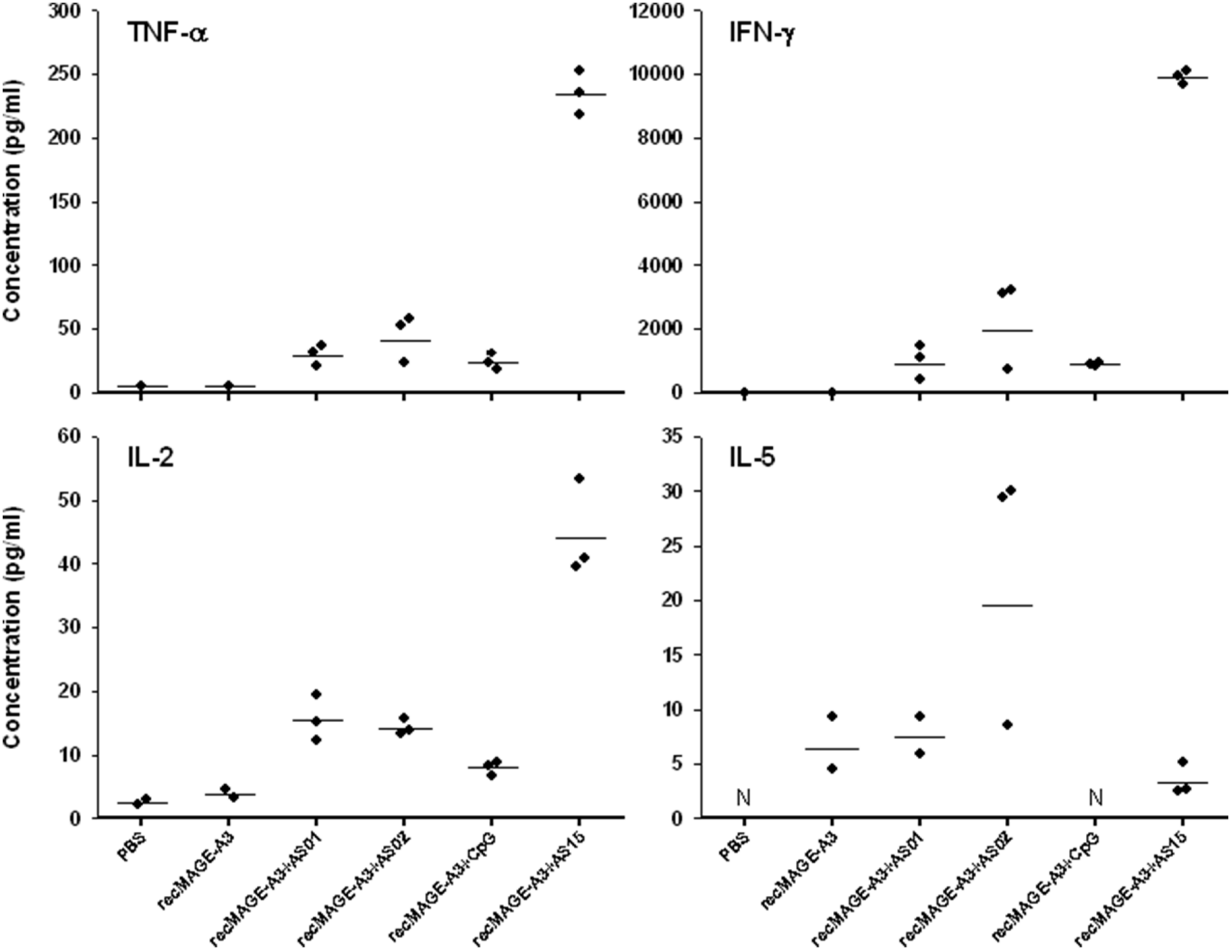
Cytokine production by isolated splenocytes from C57BL/6 mice immunized with recMAGE-A3 alone or formulated with different immunostimulants. The mice were immunized on Days 0, 14, 28 and 42 with recMAGE-A3 (10 µg of antigen) alone or recMAGE-A3 formulated with different immunostimulants, and re-stimulated *in vitro* by bacMAGE-A3. Cytokine production was measured by cytometric bead array (CBA) after 72 h of culture. Each dot represents a mouse, and bars are geomeans. N, not done.

In contrast, relevant differences between the immunostimulants were observed when the *in vitro* cytokine production by splenocytes isolated from immunized animals was measured by CBA in the culture supernatants (Figure 1). Despite the low number of mice (n = 2 or 3) in each group, our results showed that AS15 induces a clear bias towards a Th1 profile, characterized by higher IFN-γ/IL-5 and TNF-α/IL-5 ratios, comparatively to AS01, AS02 and CpG. This observation was associated with a higher production of IL-2 induced by AS15 comparatively to the other immunostimulants.

**Figure 2:**
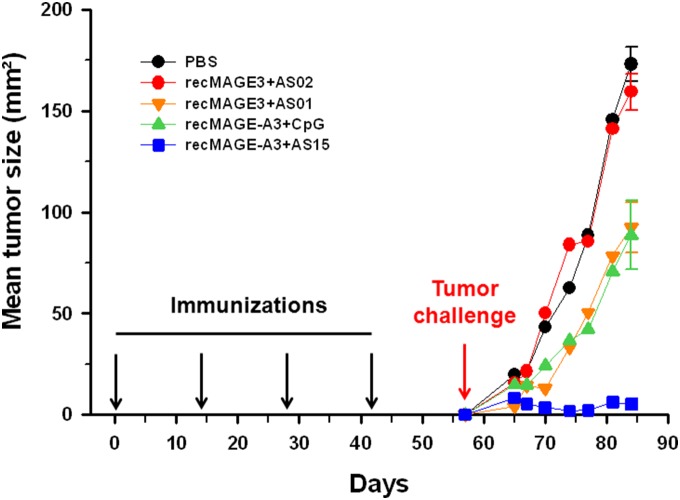
Tumor growth after tumor challenge in C57BL/6 mice immunized with recMAGE-A3 formulated with different immunostimulants. The mice (n = 5) were immunized with recMAGE-A3 (10 µg of antigen) formulated with different immunostimulants and challenged with TC1-MAGE-A3 cells. On day 84, standard errors of the mean are shown and the number of mice remaining tumor-free is indicated for each group. On Day 84, the recMAGE-A3+AS15 group was found different from any other group (p<0.01). Also, tumor growth rate was decreased in the recMAGE-A3+AS15 group, compared with the other groups (p<0.01).

After 4 immunizations with PBS or recMAGE-A3 formulated with different immunostimulants, the mice were challenged subcutaneously with TC1-MAGE-A3 tumor cells and *in vivo* tumor growth was followed during 4 weeks. In mice treated with PBS, a progressive growth of the tumors was seen (Figure 2). Different outcomes were observed for the mice immunized with recMAGE-A3, depending on the associated immunostimulant. Mice were not protected against tumor growth when AS02 was used and were poorly protected with AS01 or CpG. However, tumor growth was controlled in the mice immunized with recMAGE-A3+AS15. Not only was tumor size reduced in this group, but 3/5 mice were tumor-free when tumors were assessed four weeks after the tumor challenge.

The specificity of this anti-tumor response was established by showing that mice immunized with recMAGE+AS15 were not able to eradicate TC1 cells transfected with an irrelevant antigen (TC1-Her2/neu) injected in the same conditions as the TC1-MAGE-A3 cells (data not shown). We also observed that AS15 had to be present in every injection to efficiently stimulate anti-MAGE-A3 immunity (data not shown).

Based on the entire set of data comparing the different immunostimulants, we selected AS15 for all subsequent experiments with recMAGE-A3, as it induced a Th1-biased immune response and was the most efficient against the growth of MAGE-A3-expressing tumor cells.

### Immunization with recMAGE-A3+AS15 Elicits Long-term Protection

An important aspect in the generation of an anti-tumor immune response is the induction of long-term immune memory that is capable of providing long-term protection against tumor recurrences. In preliminary experiments in mice, we observed that increasing the number of recMAGE-A3+AS15 injections was necessary to better protect mice against the tumor challenge, suggesting that sustaining the immune response by repeated injections may be needed for improved efficacy (data not shown).

**Figure 3:**
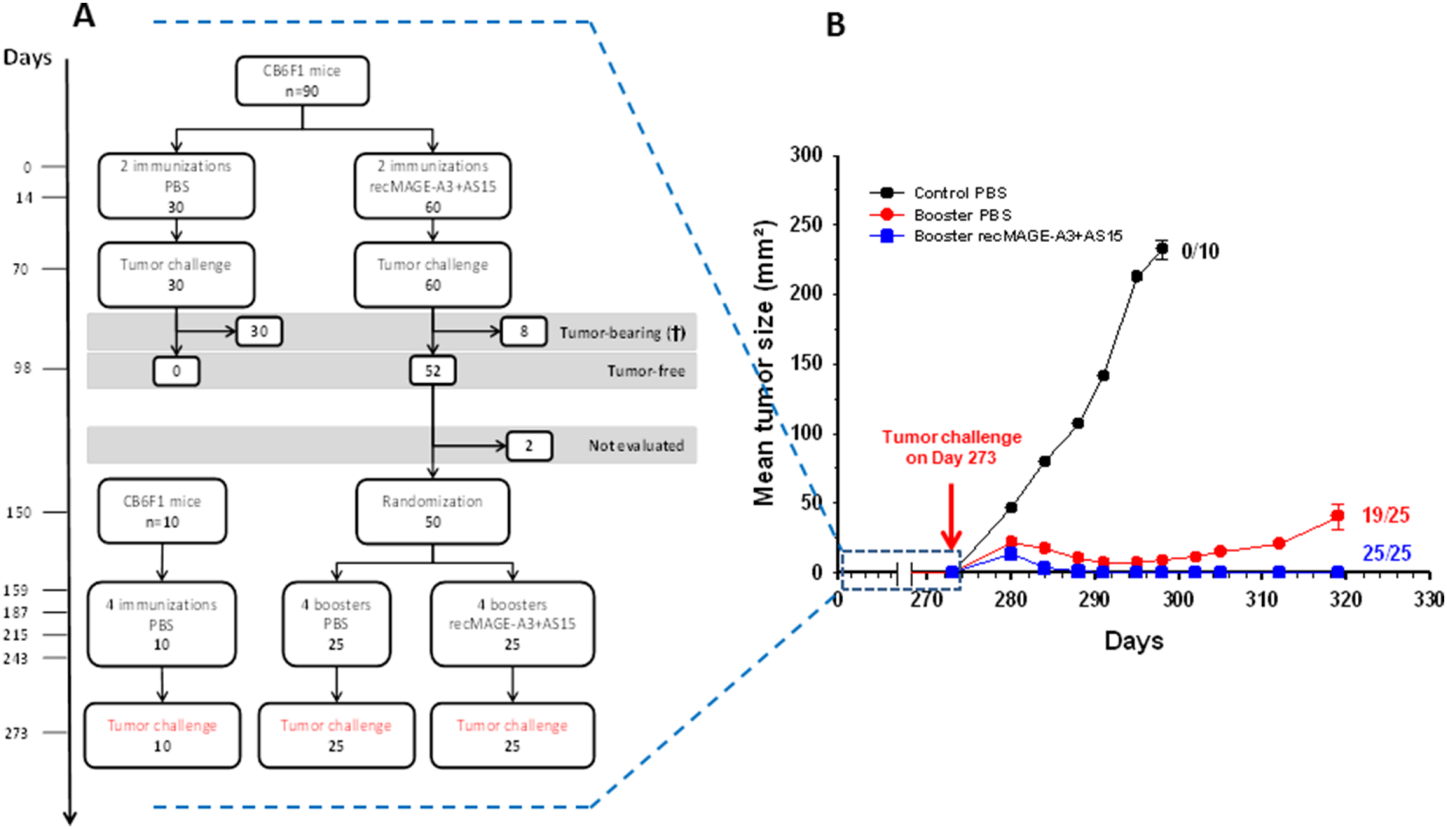
Persistence of protection after immunization with recMAGE-A3+AS15. A. Study design and sample size (CB6F1 mice) at the different steps are shown. Immunizations were made with 1 µg of antigen. B. After the second tumor challenge, tumor growth was followed for 46 days. At the end of the experiment (Day 319), standard errors of the mean are shown and the number of tumor-free mice is indicated for each group.

**Figure 4:**
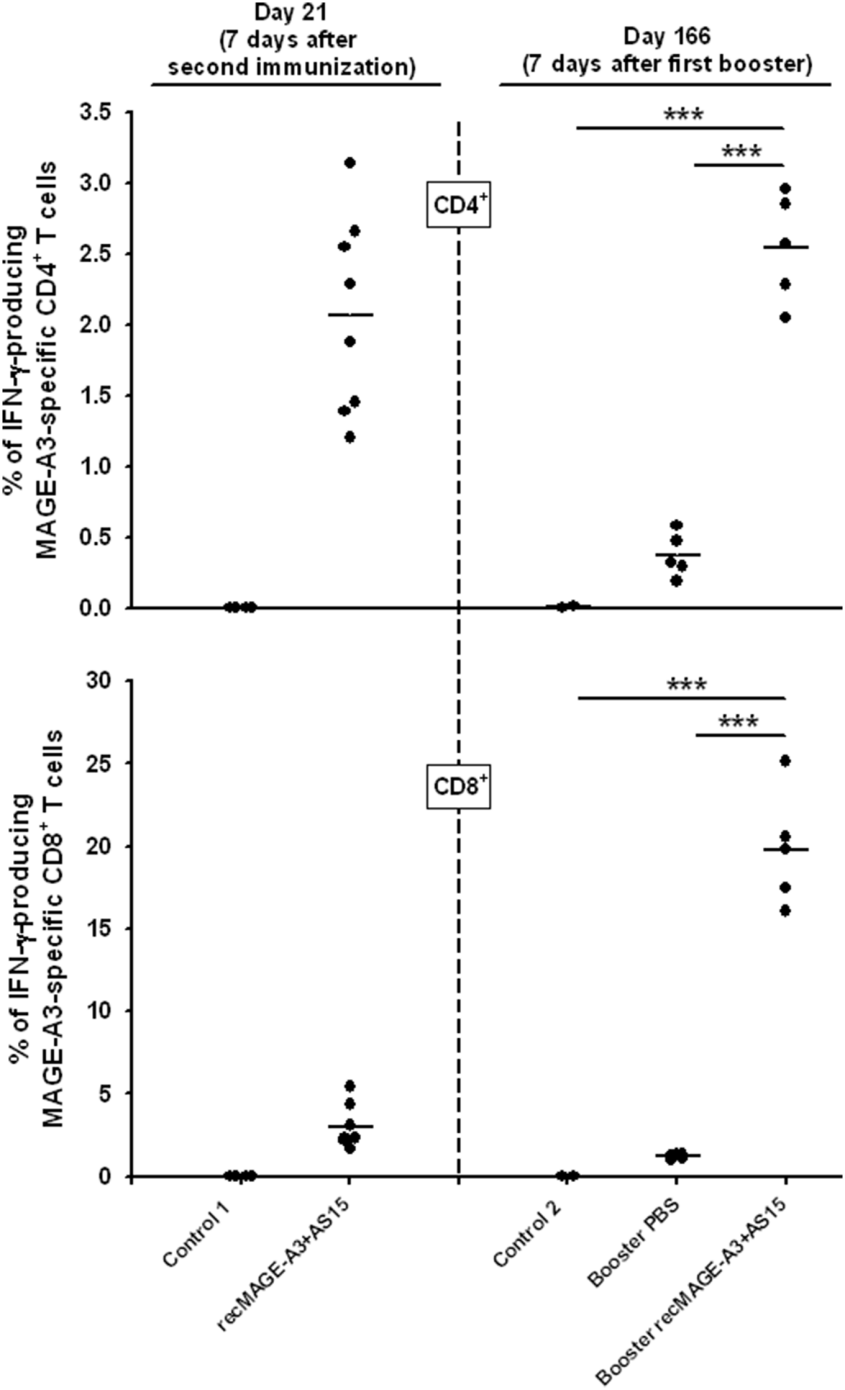
Percentage of IFN-γ-producing MAGE-A3-specific CD4^+^ and CD8^+^ T cells. CB6F1 mice were treated as shown in Figure 3A. Briefly, mice were tumor-challenged after two immunizations with recMAGE-A3+AS15 or PBS (Control 1). Mice of the MAGE-A3+AS15 group remaining tumor-free received either PBS boosters or recMAGE-A3+AS15 boosters. A new control group received PBS (Control 2). Blood samples were taken on Day 21 (7 days after the second immunization) and on day 166 (7 days after the first booster injection). Blood samples were pooled and the amounts of MAGE-A3-specific IFN- γ-producing CD4^+^ and CD8^+^ T cells were determined by intra-cellular staining and flow cytometry. Data are expressed as the percentage of total CD4^+^ and total CD8^+^ T cells after subtraction of the control medium values, which represented around 0.02% when measuring CD4^+^ T cells and 0.1% when measuring CD8^+^ T cells, respectively; each dot is a pool of 3 samples at Day 21 and each dot is a pool of 5 samples on Day 166. *** = p<0.001.

We set up an experiment to evaluate whether immunization with recMAGE-A3+AS15 was able to induce such long-term immune memory and whether boosters were necessary (Figure 3A). To this end, mice were immunized on Days 0 and 14 with either PBS or recMAGE-A3+AS15. Immunization with recMAGE-A3+AS15 induced IFN-γ-producing antigen-specific CD4^+^ and CD8^+^ T cells (Figure 4). After the challenge with TC1-MAGE-A3 tumor cells, all PBS-immunized mice developed a tumor and were sacrificed, whereas 52 of 60 recMAGE-A3+AS15-immunized mice rejected the tumor and remained tumor-free for at least two months (Figure 3A).

At this stage, 50 of the 52 tumor-free mice were randomly allocated to two groups. One group received PBS and the other group recMAGE-A3+AS15. Immune responses were evaluated at Day 166, 7 days after the first booster. In the group having received a PBS booster, the CD4^+^ and CD8^+^ T-cell responses at Day 166 (5 months after the first two immunizations with recMAGE-A3+AS15) were lower than the responses at Day 21 (one week after the first two immunizations with recMAGE-A3+AS15) (Figure 4). This illustrates the decrease in immune responses over time. In contrast, a single recMAGE-A3+AS15 booster injection was sufficient to raise the levels of cytokine-producing CD4^+^ T cells up to at least the levels measured at Day 21. In addition, the levels of CD8^+^ T cells were increased up to 5-fold compared with the levels measured one week after the first two immunizations (Figure 4) and the MAGE-A3–specific CD8^+^ T cells producing IFN-γ represented up to 20% of the CD8^+^ T cell pool.

After four monthly boosters, the 50 mice were tumor-challenged. At this stage, a third group of 10 mice receiving only PBS was introduced as a control for tumor growth. No IFN-γ-producing antigen-specific CD4^+^ and CD8^+^ T cells were detected in this control group.

In the group that received boosters of PBS, only low levels of IFN-γ-producing antigen-specific CD4^+^ T cells were observed (residual from the first two MAGE-A3+AS15 injections given 9 months earlier). However, 19 of these 25 mice remained tumor-free after the challenge, indicating that a long-term immune memory had been raised, and that mice were still protected almost one year after the last immunization. In the group of mice boosted monthly with recMAGE-A3+AS15 all 25 mice remained tumor-free (Figure 3B). These data suggest that there was a benefit of giving booster injections with recMAGE-A3+AS15, even if a long-lasting and efficient immune response was induced by the first immunization.

In a subsequent long-term experiment, we determined that AS15 was necessary in each booster to optimally protect the animals against tumor growth (data not shown).

### CD4^+^ T Cells, NK Cells, IFN-γ and MHC Class II are the Cell Subpopulations and Molecular Effectors Involved in recMAGE-A3+AS15-induced Tumor Protection

**Figure 5:**
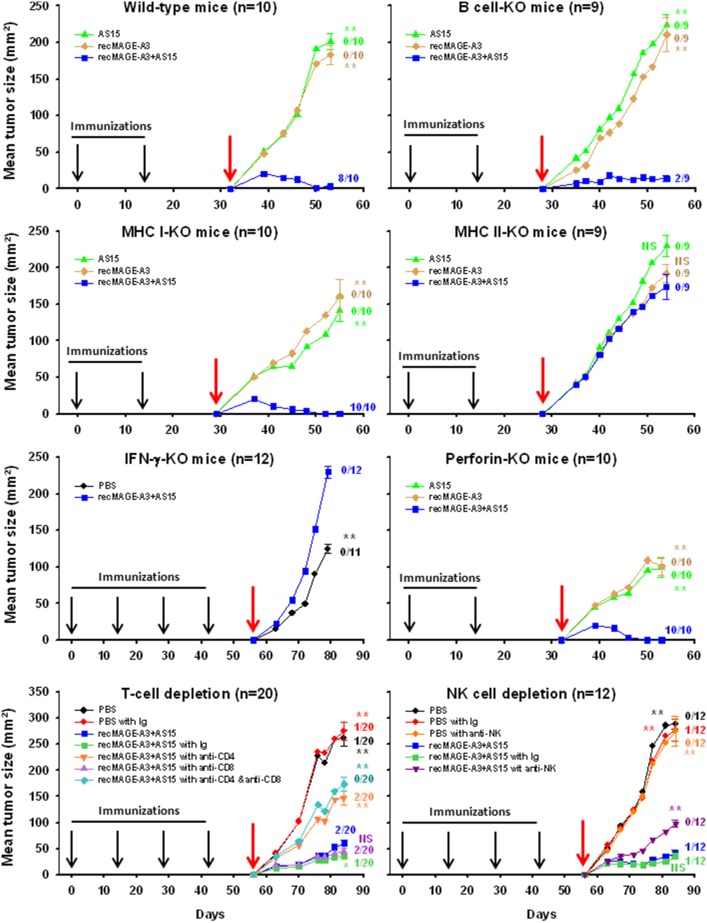
Tumour growth after tumor challenge in wild-type C57BL/6 mice, different knocked-out (KO) or cell-depleted C57BL/6 mice, immunized with either PBS, MAGE-A3 alone, AS15 alone or recMAGE-A3+AS15 (as indicated). In cell depletion experiments, control isoptypes (Ig) similar to the antibody used to deplete T cell or NK cells were used. The number of animals per group is indicated in each graph title. The red arrow indicates the time of tumor challenge. At the last time point, standard errors of the mean are shown and the number of tumor-free mice is indicated for each group. The mean tumor size of each group was statistically compared with that of the recMAGE-A3+AS15 group at the last time point (* = p<0.01; ** = p<0.001; NS = not significant).

In an attempt to identify the cells or the effector mechanisms that might be responsible for the protection against the tumor, a series of experiments was conducted in mice either KO or depleted of specific cell types. The different groups of deficient mice received two or four immunizations of recMAGE-A3+AS15 at a two-week interval before they were challenged with TC1-MAGE-A3 cells. As shown in Figure 5, B cell-KO, MHC Class I-KO and perforin-KO mice remained protected by recMAGE-A3+AS15 immunizations. In contrast, tumor protection was impacted in NK and CD4^+^ T cell-depleted mice, and in IFN-γ-KO and MHC class II-KO mice. These results identified the CD4^+^ T cells and NK cells as key cell populations in the tumor rejection mechanism. IFN-γ was also identified as a critical molecular effector. Although the exact source of IFN-γ is not known, it further emphasizes the importance of inducing a Th1-biased anti-tumor immune response.

### Tumor Growth Inhibition Depends on the Proportion of MAGE-A3-expressing Cells in the Tumor

Expression of MAGE antigens is not necessarily homogeneous in a tumor, showing focal staining in immunohistochemistry [14], probably because not all cells express MAGE-A3 at the same time and at the same level. As this phenomenon is expected to have an impact on the efficacy of recMAGE-A3+AS15 immunization, we evaluated whether recMAGE-A3+AS15 was able to protect mice against a tumor that is not composed of 100% MAGE-A3-expressing cells.

**Figure 6:**
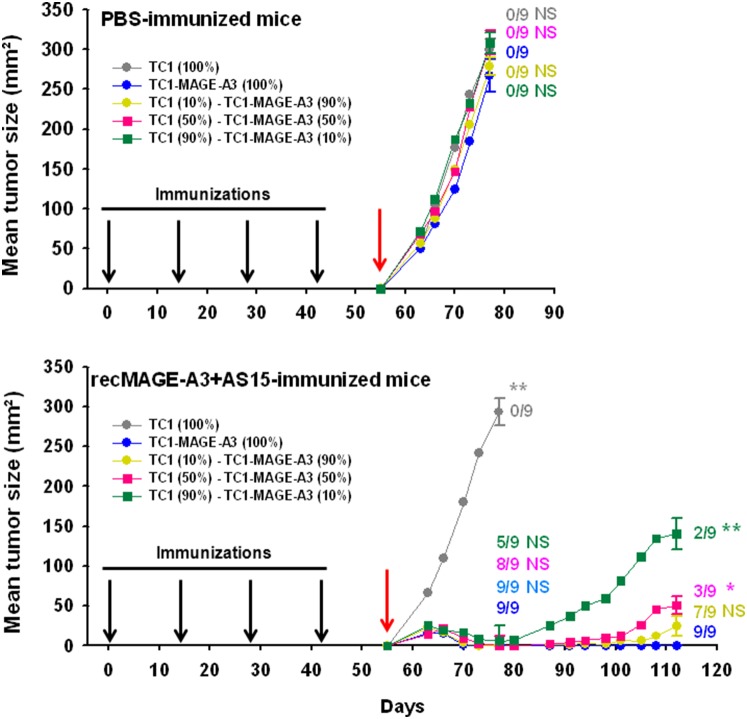
Tumor growth in C57BL/6 mice immunized with PBS (n = 9) or recMAGE-A3+AS15 (n = 9) and challenged with tumor cells containing various percentages (from 0 to 100%) of MAGE-A3-expressing cells. The mice immunized with recMAGE-A3+AS15 (1 µg of antigen) were followed up to Day 112. On Days 77 and 112, standard errors of the mean are shown and the number of tumor-free mice is indicated for each group. Statistical comparisons of the mean tumor size of each group with that the TC1-MAGE-A3 (100%) group on Days 77 and 112 are shown (* = p<0.01; ** = p<0.001; NS = not significant). Red arrow: day of challenge.

After immunization with PBS or recMAGE-A3+AS15, mice were challenged with different ratios of TC1 parental tumors and TC1-MAGE-A3-expressing cells (0–10–50–90 and 100%) (Figure 6). Results showed that all tumor mixtures grew evenly in mice sham-immunized with PBS. Likewise, the growth of a MAGE-A3-negative tumor was not impacted by recMAGE-A3+AS15 immunization. In contrast, recMAGE-A3+AS15 immunization protected against tumor growth during the 25 days following tumor challenge even when only 10% of the TC1 cells expressed MAGE-A3. The same applied when 50% of the cells, and beyond, expressed MAGE-A3. However, while all mice challenged with 100% MAGE-A3-expressing cells remained tumor-free up to 57 days after the challenge, relapses were observed in the mice challenged with tumors that contained MAGE-A3-negative cells. The intensity of the phenomenon was dependent on the percentage of MAGE-A3-negative cells in the challenging tumor. In the group challenged with 90% MAGE-A3-expressing cells, 2/9 mice showed a relapse. The mean tumor size in this group was not statistically different from that of the group receiving 100% MAGE-A3-expressing TC1 cells. In contrast, in the group challenged with 50% of MAGE-A3-expressing cells, 6/9 mice had relapsed on Day 112, and in the group challenged with 10% of MAGE-A3-expressing cells, relapses were observed in 7/9 mice on Day 112. The mean tumor size in these groups was statistically different from that of the group receiving 100% of MAGE-A3-expressing TC1 cells, with the highest mean tumor size among all relapsed animals observed in mice challenged with 10% MAGE-A3-expressing cells.

## Discussion

In the present study, we evaluated the potential of recMAGE-A3 formulated with different immunostimulants. Both the induced immune responses and their capacity to inhibit tumor growth were analyzed. For the functional experiments, the anti-tumor potential of MAGE-A3 immunizations was evaluated in a prophylactic setting with non-tumor-bearing mice rather than a therapeutic setting in order to more closely mimic the clinical situation of adjuvant treatment for cancer patients. Indeed, in the adjuvant setting, the patients first undergo surgery, and are considered free of tumor when they receive the immunization schedule. Although mouse models may not entirely reflect the human situation, partly due to intrinsic differences between the two immune systems [15] and because mice have not been primed by a primary tumor, injection of TC1-MAGE-A3 cells was selected as a tumor model to characterize the impact of the immune responses that have been induced by recMAGE-A3–based immunization. With this model, the different immunostimulants could be evaluated and at least part of the mechanisms of tumor rejection could be unraveled.

Our first and reproducible observation was that the injection of recMAGE-A3 alone did not induce protective immune responses, recMAGE-A3 being weakly immunogenic by itself. High antibody titers and detectable T-cell priming were only achieved when recMAGE-A3 was formulated with an immunostimulant. The need for recombinant proteins or peptide antigens to be mixed with immunostimulants to overcome their poor intrinsic immunogenicity is a common observation. The role of immunostimulants is essential in stimulating innate immunity and to shape the subsequent adaptive immune response [16]. This parallels other observations in clinical trials in which recMAGE-A3 was injected either alone or formulated with the immunostimulant AS02 [17].

In our study, after 4 immunizations, all immunostimulants were efficient at stimulating B cells to produce MAGE-A3-specific antibodies, as measured by enzyme-linked immunosorbent assay, and to stimulate cellular immune responses, as measured by lymphoproliferation. However, not all of the formulations performed equally in protecting the animals against a tumor challenge. Among the panel of immunostimulants tested, AS15 was the most efficient at controlling tumor size, with also a majority of animals remaining tumor-free. AS15 is the complex combination of multiple immunostimulatory molecules, targeting different immune cells. This liposome-based immunostimulant contains MPL, a detoxified derivative of LPS with TLR4 agonistic properties, QS-21, a saponin, and CpG, which is an oligodeoxynucleotide with a phosphorothioate backbone and unmethylated CpG motifs (CpG ODN 7909) that binds to TLR9. These components are potent activators of innate immunity, known to induce cellular immunity and anti-tumor immune responses [18]–[20]. MPL and QS-21 have been shown to act synergistically to induce cell-mediated immune responses [21]. Our data show that the addition of CpG to MPL and QS-21 further strengthen anti-tumor cellular immunity. Analysis of the cytokine profiles of the MAGE-A3-specific T cells revealed a peculiarity of the AS15-induced response, compared with the responses induced by the other immunostimulants. AS15 was indeed shown to induce a strong Th1-type cytokine profile, with particularly high TNF-α and IFN-γ production, two archetypal Th1 cytokines. Earlier reports on MAGE-A3- [22] or MAGE-A6-expressing tumors.

[23] highlighted the association between disease progression and Th2-polarized immune response. It was then shown that patients with active disease of any stage were skewed towards Th2-type responses against MAGE-A6 epitopes. In contrast, Th1-polarized responses were associated with no disease progression [23]. Taken together, these observations highlight the importance of a Th1-type immune response in anti-tumoral immunity, and may explain the strong protection afforded by AS15, an inducer of Th1 responses, against tumor challenge in our study. Such activity may be linked to the combined effect of CpG that stimulates plasmacytoid dendritic cells through TLR-9 activation [24], leading to enhanced T cell responses, and of the TLR-4 ligand MPL, which potentiates Th1 pathway.

By using a panel of immunodeficient mice, we tried to determine the main actors in tumor growth abrogation. Our data suggested that B cells, and thus antibody responses, were not needed to inhibit tumor growth. This might be expected as MAGE-A3 is an intracellular protein not directly accessible to antibodies for cell killing by antibody-dependent cell-mediated cytotoxicity. More surprising was the observation that CD8^+^ T cells do not seem to be essential in this *MAGEA3-*transfected tumor model, as demonstrated in CD8^+^ T cell-depleted mice or perforin-KO mice. Cytotoxic CD8^+^ T cells are often considered as the most important cell type responsible for the elimination of tumor cells. Indeed, tumor cells, being MHC class I-positive but in most of the cases MHC class II-negative, can logically only be directly targeted by the MHC class I-restricted CD8^+^ T cells. This statement has been supported by experimental results, such as the capacity of CD8^+^ T cells isolated from tumor infiltrates and passively transferred to patients after *in vitro* expansion to eliminate advanced bulky tumors [25]. In contrast, our results suggest the CD4^+^ T cells to be critical effectors in this *MAGEA3*-transfected tumor model. This is not an isolated finding, as it is in line with earlier reports showing the importance of this T-cell subset in tumor eradication in mouse models [26]–[28]. There are also findings in humans suggesting that this T-cell population plays a relevant role in tumor regression, as clinical efficacy has been reported after passive transfer of CD4^+^ T cells specific for NY-ESO-1 isolated from a melanoma tumor site [29]. The involvement of CD4^+^ T cells is not illogical, given their known central role in orchestrating the different phases of the adaptive immune response and the cross-talk they establish with other immune cells, especially the antigen-presenting cells. These actions are driven through MHC class II antigen presentation, and we observed here that MHC class II-deficient, but not MHC class I-deficient mice, cannot eradicate the tumor, which is further indication of the involvement of the CD4^+^ rather than the CD8^+^ T-cell subset. The mechanisms by which the CD4^+^ T cells may act are not clear, and different hypotheses can be put forward. Help provided by CD4^+^ T cells stimulates the expansion of a heterogeneous immune population of effector cells that are able to target different facets of tumorigenesis, acting together against tumor growth. One hypothesis relies on a CD4^+^ T cell-driven delayed type hypersensitivity (DTH)-like reaction. Antigen-presenting cells are thus attracted to the site of the tumor, capture tumor cell debris and present tumor antigens to CD4^+^ T cells through MHC class II [30]. Upon activation, T cells produce various cytokines and chemokines, attracting inflammatory cells like macrophages, granulocytes, eosinophils, and NK cells in the vicinity of the tumor [26], [31], [32]. Also particularly relevant was the observation that both CD4^+^ T cells and NK cells are necessary for an anti-tumor response [33]. Our results are in line with these observations, as there was a reduced impact on tumor growth in recMAGE-A3+AS15-immunized NK cell-deficient mice.

Among the various cytokines that can be produced by CD4^+^ cells and NK cells, IFN-γ was shown to play an essential role in tumor eradication. Indeed, IFN-γ-KO mice were unable to kill the tumor cells in our experiments. IFN-γ is produced at different stages of the immune response. It is found as early as during the initial innate response, as the result of the activation of antigen-presenting cells by certain Toll-like receptor ligands. In this regard, we have demonstrated here the capacity of AS15 to stimulate IFN-γ production. We observed measurable levels of IFN-γ in the serum of mice as early as 24 h after recMAGE-A3+AS15 injection (data not shown). IFN-γ is also produced during the development and amplification of the adaptive response. MAGE-A3-specific CD4^+^ T cells were shown to produce cytokines upon *in vitro* re-stimulation with MAGE-A3 protein or peptides. It is not the first time that a critical role is attributed to IFN-γ in controlling tumor growth (for review see [34]). Several groups have demonstrated that IFN-γ pathway-deficient mice are more prone to developing tumors [35]–[37], although the exact role played by this cytokine in tumor immuno-surveillance is not fully unraveled. IFN-γ regulates many different biological processes, and some of them may modify the tumor microenvironment, ultimately abrogating tumor growth. IFN-γ can inhibit cell proliferation [38], [39], promote apoptosis [40], [41], exert cytotoxic activity on tumor cells through the production of oxygen derivatives and nitric oxide [42], [43], and promotes the induction of inhibitors of angiogenesis by tumor cells [44], [45]. IFN-γ also has the capacity to stimulate the expression of MHC markers at the surface of malignant cells, which facilitate targeting and eradication by the host immune system (for review, see [46]). It is likely that these combined actions of IFN-γ facilitated the anti-tumor effect in our tumor challenge model. Of note, immunization with AS01 or CpG, which induced fewer IFN-γ-producing cells than immunization with AS15, did not afford full protection against a tumor challenge, highlighting a potential association between the level of IFN-γ and the level of protection.

Our work in mice demonstrated that recMAGE-A3+AS15 immunization induces a long-term immune memory, able to recognize and eliminate MAGE-A3-expressing tumor cells up to several months after the last immunization. The present study focused on the anti-tumor effect against TC1-MAGE-A3 tumors, but tumor protection was also obtained against other *MAGEA3* -transfected murine cell lines (B16-MAGE-A3 melanoma or CT26-MAGE-A3 colon carcinoma) (data not shown). Although protection was still afforded several months after the last immunization, a higher level of protection was observed if recMAGE-A3+AS15 boosters were given to sustain and even increase the levels of MAGE-A3-specific T cells. In a separate series of experiments, we showed that AS15 was necessary not only in priming, but also in booster injections. Indeed, less IFN-γ and/or IL-2-producing CD4^+^ T cells were detected, and concomitantly, less efficiency against tumor challenge was found when the booster injections were carried out without AS15 (data not shown). Similarly, an earlier MAGE-A3 study in human demonstrated the need to use an immunostimulant for priming, even if the immunostimulant is used in boosters [47]. This implies that additional spaced booster injections would be needed to sustain long-term anti-tumoral immunity, and each booster injection must be formulated with the immunostimulant in the clinical situation.

In clinical trials, patients are often enrolled based on the levels of *MAGEA3* gene expression, but the pattern of MAGE-A3 protein expression, which is not necessarily homogeneous in tumors, as demonstrated previously by immunohistochemistry [14], is not taken into account. MAGE antigens show focal expression, meaning that in an early metastasis, at the time when patients receive treatment, not all cells necessarily express MAGE-A3.

Consequently, it is reasonable to assume that tumors with a low percentage of MAGE-A3-expressing cells would be more difficult to control by anti-MAGE-A3 immune responses. We attempted to mimic this situation in mice, trying to determine the percentage of MAGE-A3-expressing tumor cells below which the elicited immune responses cannot be effective. Surprisingly, our results showed that tumor growth can still be controlled in the short term even if only 10% of cells in the tumor mass express MAGE-A3. How MAGE-A3-negative cells can be targeted after MAGE-A3 immunization is not clear. One hypothesis is that this phenomenon results from immune responses to other antigens expressed by the TC1 tumor cells, through the mechanism of antigen/epitope spreading [48], [49]. However, after several weeks, we observed a number of relapses in mice that harbored tumors with MAGE-A3-negative cells. Furthermore, the lower the percentage of MAGE-A3-expressing cells in the tumor, the higher the number of relapses and the larger the tumor size, suggesting that the phenomenon is probably due to the outgrowth of MAGE-A3-negative tumor cells. If the hypothesis is true, this also implies that the potential immune responses raised by antigen/epitope spreading would not remain efficient on the long-term, in contrast to those induced by recMAGE-A3+ AS15 immunization.

The potential of MAGE-A3 as target for cancer immunotherapy was used early in clinical trials [9], [11]. The immunostimulant in these trials was AS02, and some clinical activity was observed, especially in less advanced melanoma patients (no visceral metastasis) expressing either HLA – A1– A2 or B44. These results prompted the evaluation of recMAGE-A3+AS02 in a double-blind, randomized, placebo-controlled phase II study involving patients with completely resected MAGE-A3-expressing stage IB or II Non-Small Cell Lung Cancer (NSCLC) [10]. Another proof-of-concept phase II study was conducted in metastatic melanoma patients [8]. In this clinical study, the two immunostimulants AS02 and AS15 were compared and the results highlighted the superiority of AS15 over AS02 to elicit efficient anti-tumoral responses, with higher specific antibody titers and more robust T-cell induction. In particular, CD4^+^ T cells were shown to be major players in the observed clinical activity. This is in line with the results described in mice in the present study.

Altogether, the clinical results parallel those obtained with our tumor challenge model in mice. Our data support the use of AS15 as immunostimulant in combination with the recMAGE-A3 protein. This study highlights that pre-clinical studies are complementary to clinical development, as they can provide further information regarding the potential key effector mechanisms involved in tumor rejection and thus potentially helping in the design of recMAGE-A3-based immunotherapy in the clinical setting. Such a translational approach between preclinical and clinical data will continue to support the development of the MAGE-A3 immunotherapy, which is now under evaluation in two large, double blind, randomized phase III trials for the treatment of NSCLC (MAGRIT, NCT00480025) and melanoma (DERMA, NCT00796445).

## Supporting Information

Figure S1 **Antibody titers determined in C57BL/6 mice (n = 10 per group) immunized at 4 occasions with control saline, recMAGE-A3 alone, recMAGE-A3+AS01, recMAGE-A3+AS02, recMAGE-A3+CpG or recMAGE-A3+AS15.** Results are expressed as mid-point titers (dilution at the inflexion point of the optical density (OD)/sample dilution curve). Each dot represents a mouse and horizontal bars are geomeans. Statistical analysis: The recMAGE-A3 alone group is different from all other groups. The humoral responses induced by recMAGE-A3 formulated with an immunostimulant were considered equivalent, irrespective of the immunostimulant, as the ratio of the geometric mean titers between groups were close to 1 and all 95% CI comprised between 0.5 and 2.
(TIF)

Figure S2 **Lymphoproliferation performed on splenocytes isolated from C57BL/6 mice (n = 3) immunized at 4 occasions with control saline, recMAGE-A3 alone, recMAGE-A3/AS01, recMAGE-A3/AS02, recMAGE-A3/CpG or recMAGE-A3/AS15.** Briefly, 2×10^5^ spleen cells were plated in quadruplicate in a 96-well microplate, in RPMI medium containing 1% normal mouse serum. After 72 h of stimulation with bacMAGE-A3 (1 µg/ml), 1 µCi ^3^H thymidine was added. Sixteen hours later, cells were harvested onto filter plates. Incorporated radioactivity was counted in a β-counter and the stimulation indices were calculated. Stimulation with ConA (2 µg/ml) was included as positive control. Each dot represents a mouse and horizontal bars are geomeans.
(TIF)

Table S1 **The ARRIVE Guidelines Checklist.**
(DOCX)
